# The Effect of Flavonoids and Topiramate on Glucose Carbon Metabolism in a HepG2 Steatosis Cell Culture Model: A Stable Isotope Study

**DOI:** 10.3390/nu17030564

**Published:** 2025-01-31

**Authors:** Li Ma, Qing-Yi Lu, Shu Lim, Guang Han, Laszlo G. Boros, Mina Desai, Jennifer K. Yee

**Affiliations:** 1 The Lundquist Institute of Biomedical Innovation at Harbor-UCLA Medical Center, 1124 West Carson Street, Torrance, CA 90502, USA; mariecurie@126.com (L.M.); ghan@lundquist.org (G.H.); mdesai@lundquiast.org (M.D.); 2 Jiangsu Key Laboratory for the Research and Utilization of Plant Resources, Institute of Botany, Jiangsu Province and Chinese Academy of Sciences (Nanjing Botanical Garden Memorial Sun Yat-Sen), No. 1 Qianhuhoucun Village, Zhongshan Gate, Nanjing 210014, China; 3 Center for Human Nutrition, Department of Medicine, and Department of Pathology and Laboratory Medicine, David Geffen School of Medicine at the University of California, 10833 Le Conte Avenue, Los Angeles, CA 90095, USA; 4 Medicine and Health Care Division, Hungarian Society of Natural Sciences, Jászai Mari Square 4/a, Floor/Door 1/1, H-1137 Budapest, Hungary; contact@laszlogboros.com; 5 Department of Obstetrics and Gynecology, David Geffen School of Medicine at the University of California, Los Angeles, 10833 Le Conte Avenue, Los Angeles, CA 90095, USA; 6 Department of Pediatrics, David Geffen School of Medicine at the University of California, 10833 Le Conte Avenue, Los Angeles, CA 90095, USA

**Keywords:** hepatic steatosis, flavonoid, stable isotope, lipid, carbon flux

## Abstract

**Background**: Insufficient treatment options are available for metabolic dysfunction-associated steatotic liver disease (MASLD). Flavonoids and topiramate have been studied for weight loss but need investigation into their effects on liver metabolism. This study’s aim was to examine the effects of flavonoids or topiramate on glucose metabolic carbon flux in a cell culture model of steatosis. **Methods**: Steatosis was induced in HepG2 cells through exposure to oleic acid (OA, 0.5 mml/L) conjugated to bovine serum albumin (2:1). Additionally, 50% U^13^C-glucose was supplied in the medium as a stable isotope tracer. Cells were treated with DMSO, 10 μM of naringenin, morin, silibinin, or topiramate (44 μM) for 72 h. A non-steatotic, untreated HepG2 cell control was included. Cell extracts were analyzed by gas chromatography/mass spectrometry and mass isotopomer distribution analysis for glycogen synthesis, de novo fatty acid synthesis, tricarboxylic acid (TCA) cycle activity, and ribose synthesis. Groups were compared by ANOVA with Tukey’s pair-wise testing. **Results**: Compared to untreated HepG2 controls, OA-exposed steatotic cells exhibited increased lipid accumulation by ORO staining (1.6-fold), enhanced palmitate de novo synthesis, reduced pyruvate carboxylase/pyruvate dehydrogenase (PC/PDH) ratio, and reduced ribose synthesis. Treatment with topiramate or silibinin ameliorated the lipid accumulation (1.3-fold) and mitigated enhancement of de novo synthesis. Morin-treated cells exhibited enhanced de novo synthesis but suppressed ribose synthesis. **Conclusions**: Potential mechanisms of reduced lipid accumulation by topiramate and silibinin may include suppression of palmitate de novo synthesis and a relative decrease in carbon flux through the PDH pathway. Further studies are needed on potential utility in MASLD based on their specific metabolic effects.

## 1. Introduction

The condition previously known as non-alcoholic fatty liver disease (NAFLD) affects up to 30% of the US population [1]. Now redefined as metabolic dysfunction-associated steatotic liver disease, MASLD ranges from simple steatosis that may progress over time to metabolic dysfunction-associated steatohepatitis (MASH) with inflammation and fibrosis. The enhanced lipid accumulation in hepatocytes increases susceptibility to oxidative stress, proinflammatory cytokines, and cell death. Eventually, MASH may further progress to cirrhosis and finally hepatocellular carcinoma [2]. In addition to the liver-related morbidity and mortality, MASLD is associated with cardiovascular disease as well as T2DM, cancer, and chronic kidney disease.

Current treatment options are lacking. There is only one Food and Drug Administration-approved treatment for MASH, but no approved pharmacologic agents for earlier stages of MASLD to prevent MASH. Healthy nutrition and increased physical activity to promote weight loss remain the cornerstones of management of MASLD. In clinical care, individuals with MASLD are advised to consume a diet low in refined carbohydrates and saturated fats to reduce liver lipid production and accumulation. They are recommended to eat more fruits, vegetables, fish, and whole grains, which include antioxidant nutrients that may decrease cellular stress and inflammation. Specific foods and dietary patterns appear to be associated with better outcomes related to MASLD from systematic reviews and/or meta-analyses (SRMA). One publication that included eleven observational studies showed lower risk of MASLD among people with higher fruit and vegetable intake [3]. Another SRMA that incorporated data from nine studies demonstrated an association between coffee consumption and reduced liver fibrosis, although the general incidence of MASLD was not different [4]. Another meta-analysis that included data from four clinical trials showed improved liver function tests with green tea supplementation [5]. Therefore, research on interventions for MASLD includes the search for potential bioactive molecules found in these foods that might confer hepatoprotective effects.

Coffee, tea, and berries are food sources high in flavonoids, and these compounds have garnered much interest for their potential roles in modulating MASLD. Naringenin, derived from citrus fruit, has been shown in a number of in vitro and animal studies to modulate several biological processes implicated in MASLD, including increasing expression of peroxisome proliferator-activated receptor (PPAR)α and PPARγ and other downstream targets, regulating energy balance, improving fatty acid oxidation, modulating lipid metabolism, and reducing liver triglyceride accumulation, oxidative stress, and inflammation [6]. Silibinin, from extract of milk thistle, may antagonize the progression of MASLD by intervening in oxidative stress, insulin resistance, liver fat accumulation, and mitochondrial dysfunction [7]. A multicenter, phase III, double-blind clinical trial on 180 patients with histological diagnosis of NAFLD/NASH with or without hepatitis C demonstrated that treatment with a silybin–vitamin E–phosphatidylcholine complex for 12 months supported improvement of transaminase and γ-GT levels, along with improvement of insulin sensitivity. In 35 treated patients, a clear improvement of steatosis, lobular inflammation, ballooning, and liver fibrosis was observed compared to baseline [8]. Morin, a flavonol from mulberry and other plants, has alleviated the development of MASLD in high-fat-diet-induced obese mice, reduced body weight gain and triglyceride levels, and improved glucose tolerance, mediated by the suppression of liver X receptor (LXR) signaling [9]. Another study reported that morin mitigates reactive tumor necrosis factor-α (TNF-α) level and triglyceride accumulation in OA-induced HepG2 cells and in tyloxapol-induced mice through upregulation of PPARα and suppression of sterol regulatory element-binding protein 1c (SREBP-1c) [10]. In general, these dietary flavonoids are potent antioxidants and exhibit anti-inflammatory, antifibrotic, and antihyperglycemic effects, and therefore are potentially protective against MASLD [6,11,12].

Topiramate is an FDA-approved medication for treating epilepsy and migraines and promoting weight loss in combination with phentermine. Topiramate has a variety of physiologic effects including neurotransmitter effects through promotion of activity of the gamma-amino butyric acid (GABA)-A receptor, inhibition of glutamate receptor, and inhibition of carbonic anhydrase. Human studies have reported positively on the weight-loss-promoting and insulin-sensitizing properties of topiramate [13,14,15], in addition to improvements in circulating lipid levels. One rare case report suggested a potential role in improvement of MASLD after an adolescent lost significant body weight on topiramate and also experienced improvement in liver enzymes and hepatic steatosis. In Wistar rats fed a high-fat/high-fructose diet to induce insulin resistance with elevated ALT and glucose intolerance, animals treated with topiramate exhibited normal glucose tolerance, improvement of ALT, and increased adiponectin levels [16]. However, the study did not specifically address MASLD. Therefore, more data are needed regarding potential direct effects of topiramate on liver metabolic pathways as a potential treatment for MASLD.

Tracer-based metabolomics provides a highly comprehensive yet specific architecture to examine relationships among multiple pathways in living systems. Earlier studies using gas chromatography/mass spectrometry (GC/MS) have previously demonstrated the utility of this approach as a systems biology tool to identify perturbations of cellular metabolic pathways in response to the treatment of plant-derived bioactive small molecules [17,18,19]. Metabolites that incorporate stable isotope tracers therefore represent dynamic functional outcomes [20]. Glucose has implications in MASLD from the nutrient standpoint, serving as a substrate that donates its carbons toward multiple interconnected metabolic pathways, including glycogen synthesis, nucleotide synthesis, and lipogenesis. Therefore, use of ^13^C as a stable isotope tracer provides an opportunity to observe perturbations in the liver metabolic network over the course of an intervention. The aim of the present study was to determine the effects of three flavonoids, naringenin, morin, silibinin, and the weight-loss promoting drug topiramate on glucose-derived carbon flux in steatotic liver cell culture using U^13^C-glucose as a stable isotope tracer. We hypothesized that these compounds might promote relative pathway activities in favor of decreased liver steatosis, possibly through decreasing glucose utilization toward liver lipogenesis.

## 2. Materials and Methods

### 2.1. Chemicals

Morin (2-(2,4-dihydroxyphenyl)-3,5,7-trihydroxychromen-4-one), naringenin (5,7-dihydroxy-2-(4-hydroxyphenyl)chroman-4-one), silibinin (2,3-dihydro-3-(4-hydroxy-3-methoxyphenyl)-2-(hydroxymethyl)-6-(3,5,7-trihydroxy-4-oxobenz opyran-2-yl)benzodioxin), topiramate, glucose, and Oil Red O staining kit were purchased from Sigma-Aldrich (St. Louis, MO, USA). Naringenin and morin were also purchased from Chengdu Mansite Pharmaceutical Co, LTD (Chengdu, China). [U^13^C6]-D-glucose isotope was purchased from Cambridge Isotope Laboratories (Tewksbury, MA, USA) with 99% purity and 99% isotope enrichment for each position. Cell Counting Kit-8 assay kit, total protein assay kit (with standard: BCA method), Antibodies for Western blotting (FAS, SREBP1, PPARα) were purchased from Santa Cruz Biotechnology, Santa Cruz, CA, USA.

### 2.2. Cell Culture

**Figure 1:**
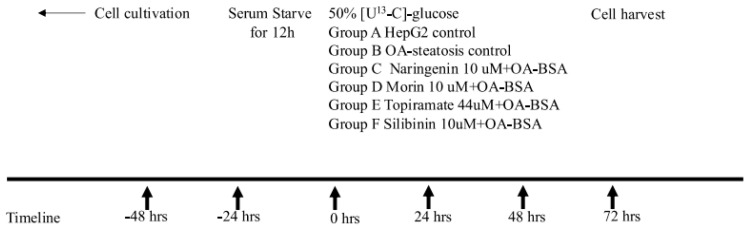
Timeline of cell culture experiment. After seeding and growing HepG2 cells in flasks, cells were serum-starved for 12 h, then were left untreated (HepG2 control) or were exposed to oleic acid (OA, 0.5 µM) conjugated to bovine serum albumin (2:1) to induce steatosis for 72 h. OA-exposed cells were also treated with one of the following compounds: DMSO (OA-exposed steatotic control), morin (10 μM), naringenin (10 μM), silibinin (10 μM), or topiramate (44 μM) for 72 h. Cell culture medium included 50% uniformly labeled glucose (U^13^C-glucose) as a stable isotope tracer and was replaced every 24 h for the duration of the experiment. At 72 h, the cells were harvested as pellets for extraction and analysis of metabolites.

Human hepatoma HepG2 cells were purchased from the American Type Culture Collection (ATCC, Manassas, VA, USA). The cells were grown at 37 °C, 5% CO_2_, and 95% humidity in Dulbecco’s modified Eagle’s medium containing 50% [U^13^C6]-glucose and 50% unlabeled glucose (100 mg/dL or 5.5 mM final concentration). HepG2 cells were serum-starved for 12 h, then were left untreated (HepG2 control) or exposed to oleic acid (OA, 0.5 µM) conjugated to bovine serum albumin (2:1) to induce steatosis. OA-exposed cells were treated with DMSO (OA-steatotic control), morin (10 μM), naringenin (10 μM), silibinin (10 μM), or topiramate (44 μM) for 72 h. The flavonoid concentrations were selected based on a literature review and were tested in our laboratory to confirm glucose utilization. A pharmacokinetic study in humans after ingestion of 135 mg oral naringenin resulted in a blood levels ranging from 7–9 μM [21]. The silibinin dose was chosen due to low or undetectable reported levels in human clinical trials and to avoid the apoptosis and reduction of glucose consumption seen in hepatocyte cell culture at a higher doses up to 200 μM [22]. Morin treatment has been published at dose ranges of 10–25 μM in hepatocyte cell cultures [9,10]. The topiramate concentration was selected based on published data using 44 uM as an approximation of physiologic concentrations ex vivo [23]. After the experimental period, cells were harvested, medium was collected, and samples were stored at −80 degree Celsius until being extracted for analysis. Figure 1 illustrates the timeline of the cell culture.

### 2.3. Lipid Droplet Staining

Lipid droplet staining was performed to quantify lipid accumulation in the cell culture model. An Oil Red O Staining Kit was used according to the manufacturer’s protocol. Cells were grown in a 24-well plate, and treated as above for 48 h. The stained cells were incubated in 250 mL 60% isopropanol and absorbance was measured at 490 nm.

### 2.4. Glucose Depletion from Cell Culture Medium

HepG2 cells were grown in high-glucose medium in 96-well plates according to the treatment groups as above. Medium was collected at 24, 48, and 72 h, and glucose concentrations were determined using glucose oxidase/peroxidase reagent (Enzymatic Assay Kits). The sensitivity of this assay was sufficient for the expected concentrations in the medium. One mL glucose oxidase/peroxidase reagent was added to ten μL cell culture medium from each sample, then incubated at 37 °C for 10 min. HPLC-grade water was used as a blank, and the control standard was glucose 5.55 mM. The plates were read at 505 nm (Molecular Devices). Glucose depletion from medium was determined by subtracting the glucose concentration from samples from the original medium concentration.

Glucose (mM) = (Au Group-Au Blank)/(Au Standard-Au Blank) × Standard mM.

### 2.5. Glycogen Production

Glucose is phosphorylated to glucose-6-phosphate which can be used toward glycogen synthesis for storage in hepatocytes. Glycogenosis has been observed in human MASLD [24]. Cell pellets were sonicated and extracted for analysis using previously published methods [25]. The isotopomer mass ratio of *m*/*z* 328 for the glucose derivative fragment was monitored and the SIGmn was calculated to represent the enrichment of glycogen from labeled glucose.

### 2.6. Fatty Acid Analysis: De Novo Synthesis, Desaturation, and Elongation

Aside from glycogen production, glucose-6-phosphate enters glycolysis to produce pyruvate, which is used as a substrate to enter the TCA cycle toward fatty acid synthesis. The GC/MS platform was used to analyze total (labeled + unlabeled) fatty acid composition, as well as incorporation of ^13^C to determine the newly synthesized fraction of fatty acids.

Cell pellets were saponified to extract fatty acids from all lipid molecular species including triglycerides, cholesterol esters, and phospholipids. Cell pellets were treated in 30% KOH (*w*/*v*) and 100% ethanol overnight at 70 degrees Celsius. Fatty acids were extracted using petroleum ether, dried under nitrogen, then were derivatized as methyl esters using 0.5 N methanolic-HCl. Analysis was performed on an Agilent 6890 N gas chromatograph coupled with a model 5975 mass spectrometer detector system. For this, 1 µL samples were injected by the automatic sampler, with each biological replicate run in duplicate or triplicate for precision. The GC column used was a Bpx70 column (30 m length × 250 µm diameter × 0.25 µm film thickness) (SGE, Inc., Austin, TX, USA). GC conditions were as follows: helium flow rate, 1 mL/min; oven temperature was initially set at 150 °C, and programmed to increase at 3 °C/min to a final temperature of 221 °C. Using known standards for comparison, palmitate was identified at a retention time of 5.1 min, palmitoleic at 5.5 min, stearate at 7.4 min, oleate at 7.9 min, vaccenate at 8.0 min, and linoleic at 8.8 min. Mass spectra were acquired using electron impact ionization and selective ion monitoring at *m*/*z* 270 for palmitate, *m*/*z* of 298 for stearate, and *m*/*z* 296 for oleate. Acetyl-CoA enrichment and de novo lipogenesis were determined from the mass isotopomer distribution of palmitate, stearate, and oleate as reported previously [26]. Peak integrations were performed on the background subtracted spectra from total ion chromatogram using Chemstation software (Agilent, Palo Alto, CA, USA).

Fatty acids produced by de novo synthesis may undergo further modifications. The relative rates of conversion over the experiment were estimated by calculating their precursor-to-product ratios [27,28]. Delta-9 desaturation of stearate to oleate is represented by the oleate/stearate desaturation index, calculated by dividing the fraction of new oleate synthesis by the fraction of new palmitate synthesis. Elongation of palmitate to stearate was calculated by dividing the fraction of new stearate synthesis by the fraction of new palmitate synthesis.

### 2.7. Glutamate

Pyruvate undergoes carboxylation into oxaloacetate for entry into the tricarboxylic acid (TCA) cycle via pyruvate carboxylase (PC) or undergoes decarboxylation via the pyruvate dehydrogenase (PDH) pathway to produce acetyl-CoA. Glutamate can be studied by isotopomer analysis for PC vs. PDH pathway contributions because it equilibrates with α-ketoglutarate, a key intermediate in the TCA cycle. ^13^C is incorporated in a signature pattern based on PC vs. PDH entry. Cell pellets underwent sonication, and extracts were precipitated in 30% ethanol. The supernatant fractions containing the amino acids were dried under a nitrogen stream. Glutamate was derivatized using n-trifluoroacetyl-n-butyl ester (TAB). GC/MS analysis was performed using a 6890 gas chromatograph and Hewlett-Packard 5973 N mass spectrometer with electron impact ionization. The GC column used was a 30 m ZB5^®^ (Phenomenex) capillary column [29]. The GC program was as follows: injector temperature 250 °C; oven temperature started at 170 °C for 2 min initially, then rose by 3 °C/min to 190 °C and 40 °C/min to an ultimate temperature of 270 °C. Helium supplied the carrier gas at 1 mL/min. The retention time of glutamate was 11.69 min. Selected ion monitoring (SIM) identified two fragments, *m*/*z* 198 and 152, corresponding to the C2–C5 and C2–C4 fragments of glutamate [30]. The isotopomer distribution was used, as previously published, to determine the pathways from which glutamate was produced [29].

Glutamate with labeled carbon at positions 4–5 results from pyruvate dehydrogenase (PDH) pathway activity, while glutamate with labeled carbon on the 2–3 positions reflects pyruvate carboxylase (PC) activity when carbon enters the TCA cycle. When both carbons are labeled on the C2–C4 fragment (m^2^ of *m*/*z* 152), this means the ^13^C was incorporated through PC, while the m^2^ isotopomer of the C2–C5 fragment (*m*/*z* 198) is evidence of ^13^C entry via PC and PDH. The pyruvate carboxylase/pyruvate dehydrogenase (PC/PDH) ratio was previously published as (m^2^ of *m*/*z* 152 fragment)/[(m^2^ of *m*/*z* 198 fragment)-(m^2^ of *m*/*z* 152 fragment)] to represent variation in pyruvate entry into the TCA cycle in the process of producing acetyl-CoA [29].

### 2.8. Cell Proliferation

Cell proliferation was determined using Cell Counting Kit-8 at 24, 48, and 72 h to determine cell survival of the treatments over the time course of the experiment. The proliferation data are complementary to the ribose synthesis data in screening for perturbations in cell replication or viability. The cells were seeded (3 × 10^3^ cells) in a 96-well plate and treated as above. Cell proliferation was detected with CCK8 reagent. Ten μL Cell Counting Kit-8 reagent was added to each well, incubated with cells at 37 °C, 5% CO_2_, and 95% humidity for 1 h, then plates were read at 450 nm with spectrophotometry (Molecular Devices SpectraMax Plus 384). Cell proliferation was calculated as a percent of the control group.

Cell proliferation (%) = A Group/A Control × 100.

### 2.9. RNA Ribose

Glucose-6-phosphate enters the pentose phosphate pathway to synthesize ribose for nucleotide production to support cellular replication. Ribose enrichment was analyzed as a reflection of cell proliferation. Trizol was used to treat cell pellets, then RNA ribose was extracted through acid hydrolysis of the chloroform-isopropanol layer. Ribose was derivatized using aldonitrile acetate, then analyzed with an HP 6890 gas chromatograph and 5973 mass spectrometer. Fragment *m*/*z* 256 (carbons 1–5 of ribose) was analyzed by mass isotopomer distribution analysis [31].

### 2.10. Western Blotting

Western blotting for SREBP1 and FAS was performed to assess for changes in lipogenic protein expression. PPARα protein expression was also investigated for potential changes in regulation of fatty oxidation. Cell pellets were sonicated in RIPA buffer with phosphodiesterase inhibitor and EDTA. Protein concentration was measured using the BCA Protein Assay (Pierce). Gel electrophoresis was performed using a precast BisTris gel (BioRad), then protein was transferred to nitrocellulose membranes. The membrane was treated with non-fat milk to block non-specific binding. Primary antibodies were applied overnight, membranes washed, and secondary antibodies applied. Bands were visualized by chemiluminescence (Pierce Super Signal West Pico Chemiluminescence kit) in a darkroom. Protein loading bands were identified by staining with Ponceau S. Densitometry was performed on the protein of interest and band intensities were normalized to the protein loading bands.

### 2.11. Statistical Analysis

SYSTAT software was used for statistical analysis. Groups were compared by ANOVA with post hoc pairwise comparisons using Tukey’s test since sample sizes in groups were similar. The Shapiro–Wilk’s test was used to confirm normal distribution of data. *p* < 0.05 was considered to indicate significant differences between treated groups compared to HepG2 control or OA-steatosis cells (steatosis control).

## 3. Results

### 3.1. Lipid Accumulation

**Figure 2:**
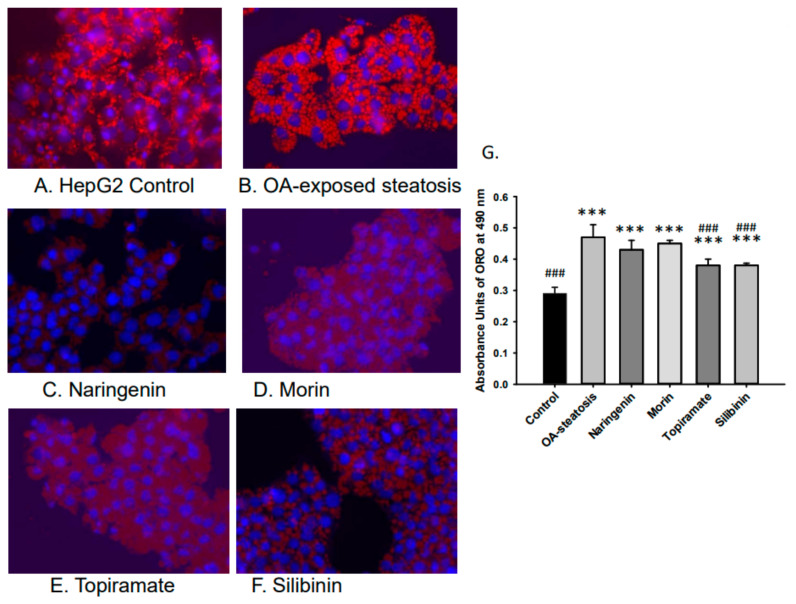
Lipid accumulation measured by absorbance of Oil Red O (ORO). Intracellular lipid accumulation was determined after ORO staining using a microplate reader. Data represents means ± standard deviation of the mean (SD) with *n* = 6 wells of cells for each group. Photos were taken at 40× magnification. (**A**). HepG2 untreated control, (**B**). OA-steatosis DMSO control, (**C**). naringenin-treated steatotic cells, (**D**). morin-treated steatotic cells, (**E**). topiramate-treated steatotic cells, and (**F**). silibinin-treated steatotic cells. (**G**). Measured absorbance units (AU) of treatment groups stained with ORO measured at 490 nm. Compared to HepG2 controls: *** *p* < 0.001. Compared to the OA-steatosis group: ### *p* < 0.001.

Lipid accumulation was studied to confirm the efficacy of OA in inducing steatosis and the relative impact of flavonoid or topiramate as interventions. Untreated cells and OA-steatotic cells with DMSO or naringenin, morin, topiramate, or silibinin were studied for lipid accumulation by ORO staining after 48 h. Overall, the groups demonstrated significant differences (*p* < 0.000001). OA-steatotic cells with DMSO showed a 1.6-fold greater intensity of staining compared to untreated controls (*p* < 0.001). Compared to the OA-steatotic cells with DMSO, treatment with naringenin or morin showed similar absorbance, whereas topiramate treatment (*p* = 0.0005) or silibinin (*p* = 0.0003) demonstrated significantly lower absorbance (1.3-fold compared to untreated non-steatotic control). (Figure 2).

### 3.2. Glucose Depletion from Cell Culture Medium

Glucose depletion was determined from medium (replaced daily) at 24, 48, and 72 h to assess for overall glucose utilization. Glucose depletion increased daily. No differences were observed among treatment groups at 24 h (aggregate mean 7.2 ± 1.8 mmol/L), or 72 h (aggregate mean 12.6 ± 2.2 mmol/L). At 48 h, differences among the groups were observed: untreated cells 11.4 ± 1.5 mmol/L, OA-exposed steatosis group 12.1 ± 1.0 mmol/L, naringenin-treated 11.5.0 ± 2.6 mmol/L, morin-treated 14.0 ± 1.7 mmol/L, topiramate-treated 11.8 ± 3.6 mmol/L, silibinin-treated 7.1 ± 3.2, *p* < 0.0015). In pair-wise testing, glucose depletion in the silibinin-treated group was significantly lower than in the OA-exposed steatotic group.

### 3.3. Glycogen Production

Glycogen synthesis is one of the three major pathway destinations for glucose-6-phosphate. Glycogenosis has been observed in MASLD in humans [24] as a sign of metabolic dysfunction, and this study examined ^13^C enrichment to detect changes in glycogen production. The isotopomer mass ratio of *m*/*z* 328 for the glucose derivative fragment was monitored and the SIGmn was calculated to represent the enrichment of glycogen from labeled glucose. At 72 h, the SIGmn was 1.65 ± 0.06 for controls, 1.55 ± 0.07 in OA-exposed steatotic cells, 1.41 ± 0.03 in steatotic cells treated with morin, 1.55 ± 0.07 in steatotic cells treated with naringenin, 1.68 ± 0.08 in steatosis cells treated with topiramate, and 1.40 ± 0.10 in steatosis cells treated with silibinin. No significant difference was identified among the groups (*p* = 0.053).

### 3.4. Fatty Acid Analysis

#### 3.4.1. Long Chain Fatty Acid Profile

**Figure 3:**
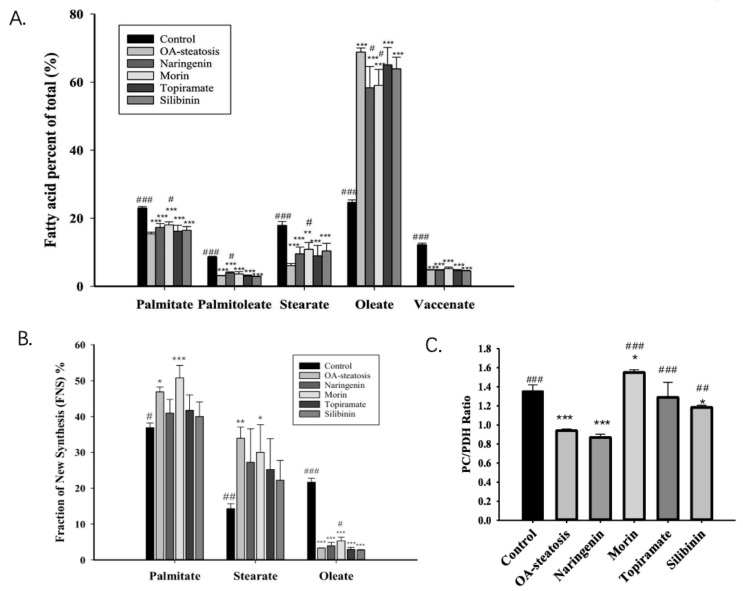
The fatty acid profile, palmitate de novo synthesis, and the pyruvate carboxylase/pyruvate dehydrogenase ratio. Fatty acids stored in liver are largely comprised of long chain fatty acids. (**A**). The abundance of the major long chain fatty acids (labeled + unlabeled) is presented as the percent of total (%) with mean and standard deviation (SD). The OA-steatotic groups exhibited oleate in the highest abundance. (**B**). The Fraction of New Synthesis (FNS), expressed as a percent, represents the de novo fatty acid synthesis rate over the 72 h experiment and is calculated from the incorporation of ^13^C. The FNS is shown for palmitate, stearate, and oleate. OA-steatotic cells demonstrated higher FNS palmitate and stearate. This difference was ameliorated by naringenin, topiramate, and silibinin. With or without treatment, OA-steatosis resulted in suppressed FNS oleate. Morin mildly raised the FNS oleate. (**C**). The pyruvate carboxylase/pyruvate dehydrogenase (PC/PDH) ratio in arbitrary units represents the relative contribution of the two pathways to de novo synthesis. Compared to HepG2 controls: * *p* < 0.05, ** *p* < 0.01, *** *p* < 0.001. Compared to the OA-steatosis group: # *p* < 0.05, ## *p* < 0.01, ### *p* < 0.001.

Fatty acids stored in liver triglycerides are largely comprised of long chain fatty acids. The total fatty acid composition includes preexisting fatty acids, those taken up from exogenous sources, and those from de novo synthesis. Cell groups were first analyzed for total long chain fatty acid composition, including labeled and unlabeled species. Figure 3A shows the major long chain fatty acids, palmitate (16:0), palmitoleate (16:1n-7), stearate (18:0), oleate (18:1n-9), and vaccenate (18:1n-7), from our samples. Linoleate, which is an essential n-6 fatty acid, had very low abundance, 1.81 ± 0.06% in HelpG2 controls and <1% in most, but not all, of the samples in the steatosis groups. n-3 fatty acids were in even lower abundance. Analysis by ANOVA showed significant differences among all groups for each of these fatty acids (*p* < 0.001). Compared to baseline control cells, OA-steatosis demonstrated decreased abundance in palmitate, palmitoleate, stearate, and vaccenate. There was an increase in abundance of oleate, which is likely due to uptake from the exogenous supply. Topiramate and silibinin both maintained the pattern seen in OA-steatosis. Compared to the OA-steatosis group, morin-treated cells had higher abundance of palmitate and lower oleate, while naringenin-treated cells had higher abundance of palmitoleate and lower oleate.

#### 3.4.2. De Novo Synthesis of Fatty Acids

Glucose-6-phosphate enters glycolysis to produce pyruvate that enters the TCA cycle to contribute toward de novo fatty acid synthesis. Palmitate (16:0) is the main product of de novo synthesis, produced from acetyl-CoA and malonyl-CoA by acetyl-CoA carboxylase and fatty acid synthase (FAS). Stearate (18:0) may be produced de novo or from chain elongation of preexisting palmitate, and oleate is produced by desaturation of stearate. The measurement of newly made fatty acids incorporating the ^13^C isotope over time is the fraction of new synthesis (FNS) expressed as a percent. ANOVA analysis determined an overall difference among the groups for palmitate (*p* = 0.00087), stearate (*p* = 0.01), and oleate (*p* < 0.001). In comparison to untreated control cells, OA-exposed steatotic cells showed an increased palmitate FNS rate (*p* = 0.002), which persisted with morin treatment. Notably, this significant increase was ameliorated in groups treated with naringenin, topiramate, and silibinin, with the FNS rates showing variation and overlap with the untreated control as well (Figure 3B). For FNS stearate, a similar pattern was shown among groups; however, a high degree of variation suggested additional factors affecting the newly synthesized stearate pool. For FNS oleate, the OA-steatosis and treatments groups all demonstrated lower rates than the untreated control. Notably, morin treatment resulted in slightly higher rates than in OA-steatosis. Acetyl-CoA enrichment was similar in all groups (baseline control 0.19 ± 0.01, OA-exposed steatosis 0.19 ± 0.00, steatotic cells treated with naringenin 0.19 ± 0.01, morin 0.19 ± 00, topiramate 0.19 ± 0.01, or silibinin 0.20 ± 0.01).

#### 3.4.3. The Desaturation Index and Elongation Index

Liver triglycerides often include at least one unsaturated fatty acid, such as oleate. Oleate is produced by delta-9 desaturation of stearate, while stearate is produced by de novo synthesis or elongation from palmitate. These processes, which impact metabolite pool size, can be reflected in the calculation of the precursor-to-product ratios. The new oleate/stearate ratio and the new stearate/palmitate ratio represent the desaturation index and elongation index, respectively (Table 1). The desaturation index was significantly different among groups (*p* << 0.001), with all steatosis groups exhibiting similarly low indices compared to the HepG2 control. The elongation index also differed among groups (*p* = 0.01). OA-steatotic cells had higher elongation indices, while naringenin maintained the higher rates. Morin, topiramate, and silibinin treatments result in variable indices that overlapped both the HepG2 control and OA-steatosis groups. Taken together, OA-induced steatosis results in suppression of delta-9 desaturation, leading to accumulation of de novo synthesized fatty acids.

#### 3.4.4. Western Blotting of SREBP1, FAS, and PPARα

**Figure 4:**
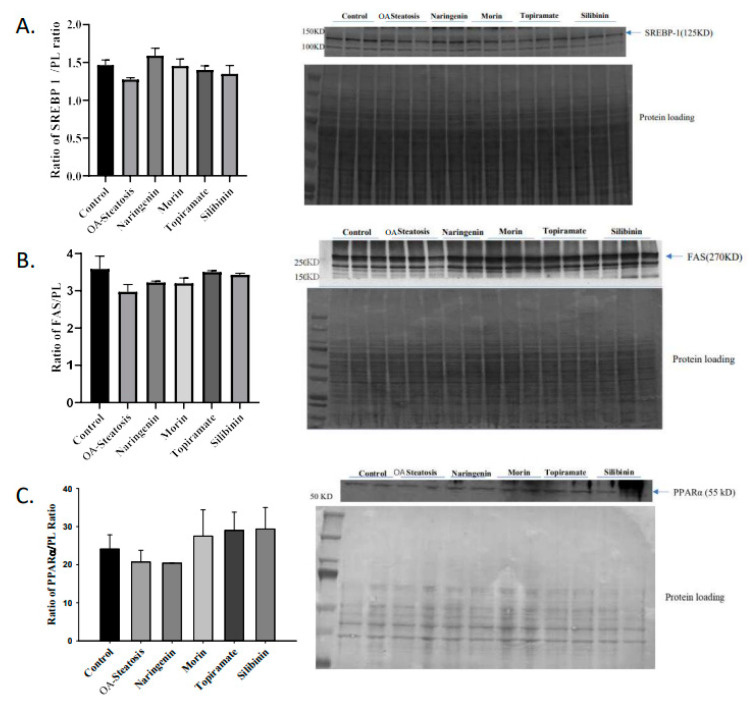
Western blotting of SREBP, FAS, and PPARα. Western blotting was performed on protein extracted from cell pellets. Signal intensity was analyzed relative to that of protein loading bands.

SREBP is a transcription factor that induces lipogenic gene expression. The enzyme FAS catalyzes fatty acid synthesis from acetyl coA and malonyl coA. Transcription factor PPARα promotes expression of genes involved in fatty acid oxidation. Western blotting was performed to determine whether protein expression of the above regulators contributed to the findings in this cell culture model. No changes in protein expression of SREBP1, FAS, or PPARα were observed (Figure 4). This leaves open the possibility of post-translational effects such as enzyme activity that contributed to the differences in de novo synthesis.

### 3.5. The Pyruvate Carboxylase/Pyruvate Dehydrogenase Ratio and TCA Cycle Activity

Pyruvate enters the mitochondria and contributes to the TCA cycle through two pathways. The pyruvate carboxylase pathway produces OAA, promotes gluconeogenesis, and counteracts oxidative stress through glutathione and NADPH synthesis. Pyruvate dehydrogenase produces acetyl-CoA for de novo synthesis. Glutamate enrichment in the 4–5 carbon positions was analyzed for pyruvate dehydrogenase (PDH) activity, while glutamate enriched on the 2–3 carbon positions was analyzed for pyruvate carboxylase (PC) activity for entry into the TCA cycle. The PC/PDH ratio is a reflection of the relative contribution of each pathway to the TCA cycle. Compared to untreated control cells (1.35 ± 0.07), OA-induced steatosis reduced the ratio, (0.94 ± 0.02, *p* < 0.001). In comparison to the OA-steatosis group, the ratio was maintained by naringenin treatment (0.87 ± 0.03). whereas morin (1.55 ± 0.03, *p* < 0.001), topiramate (1.29 ± 0.16, *p* < 0.001), and silibinin (1.18 ± 0.02, *p* = 0.002) promoted higher ratios (Figure 3C).

### 3.6. Cell Proliferation

The groups of cells showed no differences among them and exhibited full proliferation at each time point. At 72 h, values were control 100.0 ± 1.2%, OA-steatosis 100.0 ± 1.3%, naringenin 100 ± 3.9%, morin 100 ± 2.9%, topiramate 100 ± 9.9%, and silibinin 100 ± 2.3%.

### 3.7. RNA-Ribose Synthesis

**Figure 5:**
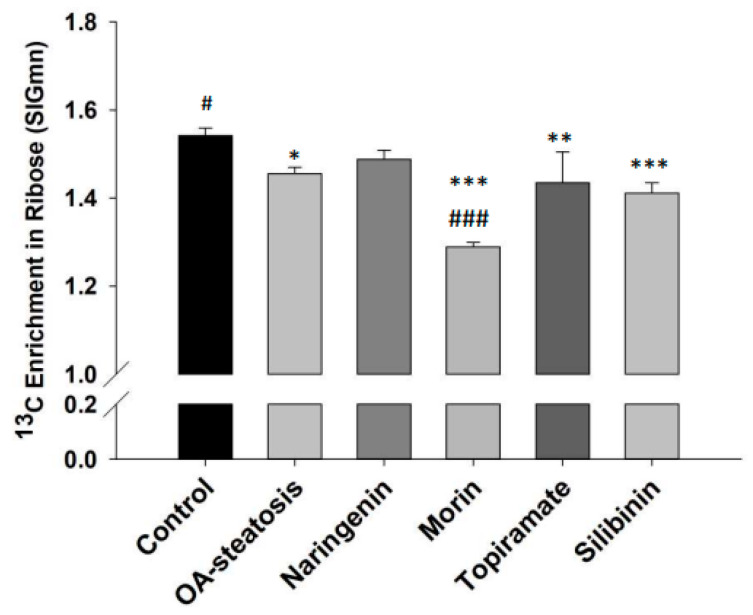
^13^C Enrichment in ribose (SIGmn). Glucose-6-phosphate enters the pentose phosphate pathway (PPP) for production of nucleotides to support cell replication. Ribose production reflects PPP activity. The ^13^C molar enrichment in ribose represents ribose synthesis over the time course of the experiment. In OA-steatosis, ribose synthesis is lower than when compared to the non-steatotic untreated control (*p* = 0.002). Compared to HepG2 controls: * *p* < 0.05, ** *p* < 0.01, *** *p* < 0.001. Compared to the OA-steatosis group: # *p* < 0.05, ### *p* < 0.001.

One of the three major pathways of entry by glucose-6-phosphate is the pentose phosphate pathway (PPP). The PPP produces nucleotides necessary for cell replication activities. The incorporation of ^13^C from glucose reflects RNA-ribose new synthesis activities. The contribution of glucose carbon to ribose is calculated by determining the ^13^C molar enrichment in ribose (Figure 5). OA treatment (1.46 ± 0.001) significantly lowered ribose synthesis when compared to the untreated control (1.54 ± 0.001, *p* = 0.002). Compared to the OA-steatotic cells, silibinin (1.41 ± 0.001) and topiramate (1.43 ± 0.001) did not change the rate, while morin further lowered it (1.29 ± 0.03, *p* < 0.001). Naringenin (1.49 ± 0.03) enrichment was not different from either control cells or the OA-steatosis cells.

### 3.8. Results Summary

**Figure 6:**
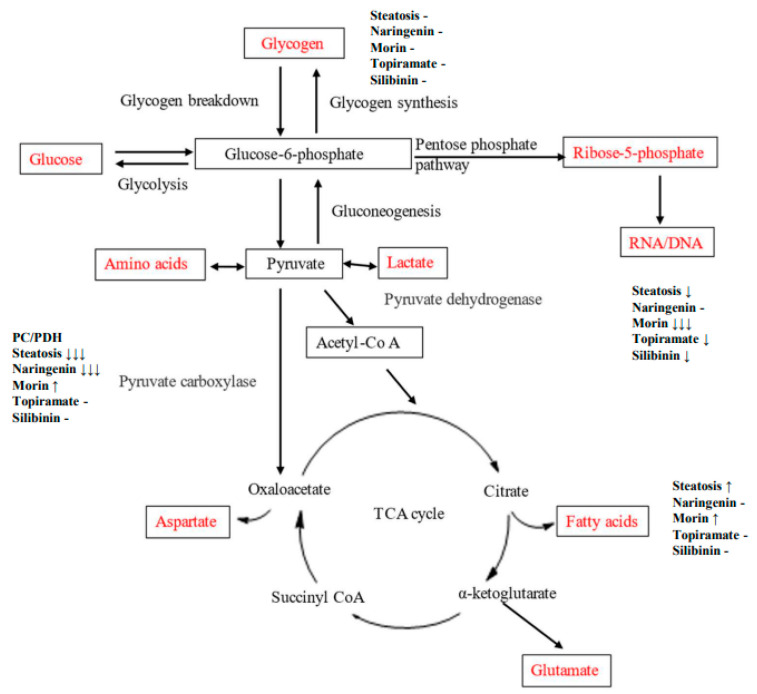
Diagram of metabolic pathways through which carbon flux occurs. Direction of change in OA-steatotic cells and their treatment groups is indicated at each measured metabolic outcome, relative to non-steatotic, untreated HepG2 cells.

An OA-induced steatosis cell culture model was created in HepG2 cells to study the contribution of glucose to carbon metabolic pathways and impact of flavonoids or topiramate as interventions. The steatotic cells demonstrated lipid accumulation by ORO and robust cell proliferation. Using U^13^C-glucose, relative pathway analysis of outcomes from the three major destinations for glucose-6-phosphate were studied, showing that OA-steatosis was associated with lower ribose synthesis from the PPP, no change in glycogen synthesis, enhanced new synthesis of palmitate and stearate, increased elongation, and lower desaturation to oleate. (Figure 6, Table 2). Interventions showed some mitigation of lipid accumulation by topiramate and silibinin and mitigation in enhancement of de novo synthesis and the PC/PDH ratio. Naringenin treatment resulted in persistently enhanced elongation and improved ribose synthesis. Morin-treated cells showed enhanced de novo synthesis of saturated fatty acids, as well as oleate.

## 4. Discussion

This study was designed to investigate the effects of three flavonoids and topiramate on glucose-derived carbon flux in a HepG2 cell culture model of steatosis. We examined outcomes related to three main pathways through which glucose enters as glucose-6-phosphate: glycogen synthesis, the pentose phosphate pathway, and glycolysis, which produces pyruvate to enter the mitochondria into the TCA cycle. For the interventions, unlike many in vitro studies that have used high doses, we used relatively low doses (flavonoids 10 µM, topiramate 44 µM) that are physiologically relevant to the concentrations found in human blood after ingestion. For example, serum concentrations of naringenin in healthy subjects who ingested a single dose of 150 mg naringenin were found to be 15.76  ±  7.88 μM [21].

Oleic and palmitic acid are the most abundant fatty acids in hepatic triglycerides. Change in fatty acid composition through de novo synthesis or turnover may contribute to the pathogenesis of MASLD [32]. Oleic acid has been shown to be more steatotic than palmitic acid [33] and to promote reactive oxygen species (ROS) production and TNFα expression [34], which contribute to the progression of MASLD to MASH. OA-exposed HepG2 hepatocytes were often employed as an in vitro model of steatosis to investigate the molecular mechanisms underlying MASLD [34,35]. Results from our study indicated that OA-exposed cells significantly increased intracellular lipid accumulation, consistent with published data [35], and demonstrated ample cell proliferation. Relative pathway analyses in OA-steatotic cells showed no effect on glycogen synthesis, with lower ribose synthesis rate but higher de novo synthesis. Together with higher elongation and suppressed desaturation, the fatty acid pathways are shifted toward synthesis and accumulation of new saturated fatty acids, promoting a lipid composition for storage. A similar shift was previously reported in an alcohol-induced rat model of liver disease [28], supporting triglyceride storage and reduced export. The lower PC/PDH ratio suggests a relative increase in PDH activity.

The observation of the lower PC/PDH ratio in our OA-steatotic cells contrasts with previously published studies on the PC and PDH activities in MASLD. Mice with high-fat-diet-induced hepatic steatosis demonstrated benefits from having enhanced PDH activity from inhibition of pyruvate dehydrogenase kinase 2 and decreased PC flux through increased fatty acid oxidation and ketone body formation [36]. A tracer study on TCA cycle activity in humans with MASLD supported higher activity through PC [37]. Therefore, we speculate that our finding of lower PC/PDH might reflect a metabolic adaptive response under our artificial cell culture system rather than intrinsic dysfunction in rodent models and in human disease.

In our study, we found that silibinin exposure significantly reduced lipid accumulation in OA-steatotic HepG2 cells. Silibinin-treated cells did not demonstrate the increase in de novo synthesis rate that was observed in untreated OA-steatotic cells. These results suggest that silibinin could potentially ameliorate lipid accumulation induced by OA in HepG2 cells. The increase in PC/PDH ratio with sibilinin treatment suggests a restoration of the balance between the two pathways. Although SREBP1, FAS, and PPARα protein expression appeared unchanged in all groups including silibinin, the protein expression is a static marker of activity at the end of the experiment, whereas the FNS represents the accumulation of de novo synthesis over the course of 72 h. Silibinin treatment maintained the diminished ribose synthesis seen in OA-steatotic cells, which suggests an inability to correct nucleotide imbalance. A reciprocal relationship between the inhibition of the de novo lipogenesis (palmitate synthesis) and the inhibition of ribose/deoxyribose has been noted in several studies [17,31,38].

Silibinin is the only compound investigated that demonstrated decreased glucose depletion from the medium, but only at the 48 h time point. The dose of silibinin used in this experiment was relatively low compared to published data showing that silibinin treatment (20–100 µM) increases glucose uptake and decreases intracellular triglyceride in OA-treated HepG2 cells [39]. The published study also demonstrates that silibinin can ameliorate some metabolic alterations and induce some molecular changes by activating the Caspase 8 and Fas-associated protein with death domain-like apoptosis regulator and c-Jun N-terminal kinase (CFLAR-JNK) pathway, thereby regulating its downstream target genes involved in lipid metabolism (PPARα, SREBP-1c, and PNPLA3), glucose uptake (PI3K-AKT), oxidative stress (NRF2, CYP2E1, and CYP4A), and inflammatory response (NO) in OA-treated HepG2 cells. Most of these effects were observed at doses of 50 µM or higher [35], so dose-dependent variation is possible. The overall profile of silibinin has also been described as a mimetic for the fasting state through promotion of the AMPK pathway and inhibition of glycolysis [22].

Treatment with other flavonoids impacted metabolic pathways differently, and with less success in lipid accumulation. Although a published study by Gu et al. found that morin mitigated triglyceride (TG) accumulation in OA-induced HepG2 cells [9], morin did not overtly affect lipid accumulation in our study, possibly due to the relatively low dose used. On the other hand, in comparison to the OA-steatosis group, morin treatment notably maintained similarly high FNS palmitate and stearate rates, but this was also the only intervention group to demonstrate a higher FNS oleate. Despite appearing very lipogenic, the restoration of PC/PDH to be similar to HepG2 controls suggests some improvement of phenotype mediated by morin. A benefit of the PC pathway includes protection from oxidative stress through glutathione synthesis, which removes ROS that may cause inflammation and cell damage. A published hepatocyte cell culture study on glucotoxicity-induced liver damage showed that morin was protective through reduction of ROS [40]. Whether morin’s antioxidant effects are attributable to PC activity in MASLD requires future research. On the other hand, naringenin promoted ribose synthesis, but maintained a lower PC/PDH ratio similar to the OA-steatosis group. It is possible that naringenin is protective against hepatocyte steatosis via enhancing ribose synthesis to support nucleotide balance. Naringenin may have potential benefits in pathways not studied here. In mice with MASLD induced by methionine-choline deficiency and in HpeG2 cells treated with OA, naringenin reduced lipid accumulation through inhibition of the inflammatory cytokine-mediated signaling pathway NLRP3/NFkB [41].

Topiramate is a potent antiepileptic drug whose side effects of weight loss in patients with epilepsy and obesity led to the FDA approval of this drug combined with phentermine as an anti-obesity medication. Topiramate has been studied more for its insulin sensitizing and weight control properties, and less is known about its effects on MASLD. A study of the effects of topiramate in female Zucker diabetic fatty (ZDF) rats demonstrated that topiramate treatment leads to a decrease in hepatic glucose output, increased insulin sensitivity in adipose but not muscle, and suppression of adipose tissue lipolysis [23]. The study focused largely on actions in adipocytes and muscle. Although MASLD was not studied explicitly, topiramate treatment in rats with high-fat/high-fructose-induced insulin resistance resulted in increased liver expression of adiponectin receptors, GLUT2, and tyrosine kinase activity [16], thereby implicating pathways of glucose utilization and cell proliferation. Our study contributes toward filling the knowledge gap regarding topiramate’s effects on MASLD, showing that topiramate may counteract OA-steatosis by inhibiting lipid accumulation, in association with some abatement of enhanced de novo synthesis and improvement in the PC/PDH balance.

Limitations of this study include the use of single doses of each compound, so information was not obtained on dose-dependent effects. Use of U^13^C-glucose as the tracer did not permit separate examination of the transketolase and transaldolase pathways in the ribose analysis. Western blotting determined protein expression only, which leaves open the possibility of post-translational effects. This study focused on carbon metabolism derived from glucose and does not address all systems affected by steatosis (e.g., ER stress and inflammation) or potential interplay between organ systems. Cell culture systems such as this OA-induced model do not completely replicate the physiology of MASLD seen in human subjects.

## 5. Conclusions

In this OA-induced HepG2 cell culture model of liver steatosis, glucose utilization was associated with decreased ribose synthesis, enhanced de novo synthesis, and suppression of desaturation to oleate, all reflecting relative pathway activities favoring lipid storage. A ^13^C tracer analysis allowed the detection of small but significant differences in pathway activities among cells treated with flavonoids or topiramate. Silibinin and topiramate decreased lipid accumulation and mitigated enhancement of de novo synthesis. Morin treatment appeared to favor de novo synthesis pathways, drawing glucose-derived carbons aways from ribose synthesis. Since the doses used were based on levels relevant to human exposure, the mild effects observed in our study could be consistent with the variation of effects in human disease. Future preclinical or clinical studies could explore how short-term vs. long-term treatment affects these pathways. Additional studies would be useful to investigate whether the treatment is effective at various stages of MASLD, including for MASLD prevention and the more advanced stages leading up to MASH.

## Figures and Tables

**Table 1:** Desaturation and elongation. The ratio of new oleate/stearate represents delta-9 desaturation of stearate to produce monounsaturated oleate. The ratio of new stearate/palmitate reflects chain elongation of 16-carbon palmitate to 18-carbon stearate. Ratios are shown as mean values with the standard deviation. Compared to HepG2 controls: * *p* < 0.05, ** *p* < 0.01, *** *p* < 0.001. Compared to the OA-steatosis group: ## *p* < 0.01, ### *p* < 0.001.

Fatty Acid Ratio	Control	OA-Steatosis	Naringenin	Morin	Topiramate	Silibinin
Oleate/stearate desaturation index	1.53 ± 0.14 ^###^	0.10 ± 0.01 ***	0.17 ± 0.09 ***	0.19 ± 0.07 ***	0.13 ± 0.07 ***	0.13 ± 0.03 ***
Stearate/palmitate elongation index	0.39 ± 0.02 ^##^	0.72 ± 0.05 **	0.65 ± 0.16 *	0.59 ± 0.10	0.59 ± 0.16	0.55 ± 0.09

**Table 2:** Summary of changes noted in metabolic pathways studied. The untreated HepG2 group is the reference control group.

Metabolic Pathway	Control	OA-Steatosis	Naringenin	Morin	Topiramate	Silibinin
Glycogen	reference	-	-	-	-	-
Palmitate and stearate de novo synthesis	reference	increased	-	increased	-	-
Delta-9 desaturation	reference	decreased	decreased	decreased	decreased	decreased
Elongation	reference	increased	increased	-	-	-
PC/PDH ratio	reference	greatly decreased	greatly decreased	increased	-	-
Ribose synthesis	reference	decreased	-	greatly decreased	decreased	decreased

## Data Availability

The dataset is available on request from the authors.

## Abbreviations

The following abbreviations are used in this manuscript:

OA	Oleic acid
MASLD	Metabolic dysfunction-associated steatotic liver disease
MASH	Metabolic-dysfunction-associated steatohepatitis
TCA	Tricarboxylic acid cycle
PC/PDH	Pyruvate carboxylase/pyruvate dehydrogenase
